# Germination of *Aspergillus fumigatus* inside avian respiratory macrophages is associated with cytotoxicity

**DOI:** 10.1186/1297-9716-43-32

**Published:** 2012-04-19

**Authors:** Lieven Van Waeyenberghe, Frank Pasmans, Katharina D’Herde, Richard Ducatelle, Herman Favoreel, Shao-Ji Li, Freddy Haesebrouck, An Martel

**Affiliations:** 1 The Department of Pathology, Bacteriology and Avian diseases, Faculty of Veterinary Medicine, Ghent University, Salisburylaan 133, B-9820, Merelbeke, Belgium; 2 The Department of Basic Medical Sciences, Faculty of Medicine and Health Sciences, Ghent University, De Pintelaan 185, B-9000, Ghent, Belgium; 3 The Department of Virology, Parasitology and Immunology, Faculty of Veterinary Medicine, Ghent University, Salisburylaan 133, B-9820, Merelbeke, Belgium

## Abstract

Although aspergillosis is one of the most common diseases in captive birds, the pathogenesis of avian aspergillosis is poorly known. We studied the role of avian respiratory macrophages as a first line of defense against avian aspergillosis. The phagocytic and killing capacities of avian respiratory macrophages were evaluated using pigeon respiratory macrophages that were inoculated with *Aspergillus fumigatus* conidia. On average, 25% of macrophage-associated conidia were phagocytosed after one hour. Sixteen percents of these cell-associated conidia were killed after 4 h and conidial germination was inhibited in more than 95% of the conidia. *A. fumigatus* conidia were shown to be cytotoxic to the macrophages. Intracellularly germinating conidia were located free in the cytoplasm of necrotic cells, as shown using transmission electron microscopy. These results suggest that avian respiratory macrophages may prevent early establishment of infection, unless the number of *A. fumigatus* conidia exceeds the macrophage killing capacity, leading to intracellular germination and colonization of the respiratory tract.

## Introduction

Fungal infections caused by *Aspergillus* species are a major cause of morbidity and mortality among captive birds [1]. The filamentous and saprophytic *A. fumigatus* is the predominant species found in infected birds [2]. This fungus produces huge numbers of airborne conidia, which are found ubiquitously in the environment [3].

The primary route of infection in birds is inhalation of these airborne conidia, resulting in lower respiratory tract colonization [4]. In mammals, the alveolar macrophage is the first line of defense against inhaled conidia [5-7]. In vitro studies show that alveolar macrophages phagocytose and kill attached conidia [6,8-10]. However, in rabbits and mice, conidia of *A. fumigatus* occasionally seem to be able to germinate within alveolar macrophages [11,12].

In comparison to the mammalian respiratory system, a paucity of free respiratory macrophages exists in the avian respiratory system [13-16]. However, scarcity of free respiratory macrophages does not necessarily mean that the avian pulmonary defenses are compromised. Avian respiratory macrophages are innately more competent in phagocytosing particles than mammalian alveolar macrophages and an efficient translocation of subepithelial macrophages to the epithelial surface has been demonstrated [17,18]. Moreover, the epithelium and the interstitial macrophages of the atrial and infundibular area play an important role in the removal of particles from the air [16,19].

Due to predominant colonization of the avian lower respiratory tract, avian respiratory macrophages can fulfill an important defensive role during the initial colonization events. Therefore, the aim of this study was to assess the role of the avian respiratory macrophage in avian aspergillosis.

## Material and methods

### *A. fumigatus* growth conditions

The *A. fumigatus* isolate, K24, used in the in vitro studies, was obtained from a racing pigeon, which died from pulmonary aspergillosis [20]. Five-day-old cultures of this isolate on Sabouraud dextrose agar (CM0041, Oxoid Ltd., Basingstoke, England) were washed with 5 mL of 0.01% Tween 20 in RPMI 1640 to harvest *A. fumigatus* conidia. The conidia were washed three times in 0.01% Tween 20 in RPMI and the suspension was adjusted to a concentration of 10^6^*A. fumigatus* conidia/mL in RPMI 1640 with 1% glutamine and 1% pyruvate by haemocytometer count.

### Cells

Pigeon respiratory macrophages were collected according to a method as previously described [21], and maintained in RPMI 1640 supplemented with 1% glutamine and 1% pyruvate. Cell sample purity was determined using non specific esterase (Sigma Diagnostics, St. Louis, MO, USA) and Haemacolor (Merck, Darmstadt, Germany) staining.

### Conidiacidal ability of pigeon respiratory macrophages

To assess the killing capacity of avian respiratory macrophages against *A. fumigatus* conidia, pigeon respiratory macrophages were counted and seeded into a 96-well plate at 10^5^ macrophages per well. After 2 h of incubation at 37°C and 5% CO_2_, the wells were rinsed to remove non adherent cells. The pigeon respiratory macrophages were exposed to 0.2 mL of 10^6^ conidia/mL in RPMI 1640 (+ 1% glutamine and 1% pyruvate). The plates were centrifuged at 125 × *g* for 10 min to synchronize conidial exposure to the cells. Subsequently, the cells were allowed to ingest the conidia for 1 h at 37°C, 5% CO_2_. Medium containing non adherent, non phagocytosed conidia was removed, and wells were rinsed three times using HBSS with Ca^2+^ - Mg^2+^ at 37°C and fresh medium was added (time point 0). The macrophages were then further incubated for 4 h before cell associated conidia were harvested (time point 4). At time points 0 or 4, plates were frozen at -70°C and rapidly thawed at 37°C to lyse the cells and harvest the conidia. Cellular lysis was confirmed by light microscopy. Serial dilutions were performed in sterile medium and immediately plated on Sabouraud dextrose agar. Plates were incubated at 37°C, and colonies were counted after 24 h of incubation. Negative control wells did not contain macrophages but were otherwise treated likewise. The percentage killed conidia was calculated by dividing the total number of conidia recovered at 4 h by the total number of conidia recovered at 0 h and multiplied by 100. All tests were performed in triplicate.

### Phagocytic capacity of pigeon respiratory macrophages

To assess the percentage phagocytosed *A. fumigatus* conidia, pigeon respiratory macrophages were seeded in 24-well plates and exposed to 1 mL of 10^5^ conidia/mL. The plates were centrifuged at 125 × *g* for 10 min to synchronize conidial exposure to the cells. After phagocytosis for 1 h, medium containing non adherent, non phagocytosed conidia was removed and the wells were rinsed three times using HBSS with Ca^2+^ - Mg^2+^ at 37°C. To discriminate between ingested and adherent conidia, the cells were further incubated with Calcofluor White M2R (Life Technologies Europe BV, Ghent, Belgium), which stains the fungal cell wall of extracellular, but not that of intracellular conidia. For quantification of phagocytosis, the cells were then washed and fixed with 4% (v/v) para-formaldehyde for 10 min at room temperature. Per well, 100 conidia were analyzed for intra- vs. extracellular location using fluorescence microscopy. All tests were performed in triplicate.

### Intracellular germination assessed by a fluorescence assay and transmission electron microscopy

To quantify intracellular germination, pigeon respiratory macrophages were seeded in 24-well plates and exposed to 1 mL of 10^5^ conidia/mL. The plates were centrifuged at 125 × *g* for 10 min to synchronize conidial exposure to the cells. After phagocytosis for 1 h, medium containing non adherent, non phagocytised conidia was removed and the wells were rinsed three times using HBSS with Ca^2+^ - Mg^2+^ at 37°C. To discriminate between ingested and adherent conidia, the cells were further incubated with Calcofluor White M2R. The cells were further incubated for 6 h and then fixed with 4% (v/v) para-formaldehyde for 10 min at room temperature. Per well, 100 conidia were analyzed for germination activity using fluorescence microscopy. The tests were performed in triplicate. To observe the intracellular trafficking and intracellular germination of *A. fumigatus* conidia electron microscopically, pigeon respiratory macrophages were seeded in 24-well plates at 5 × 10^5^ / mL and incubated for 2 h at 37°C and 5% CO_2_. After the incubation period, the wells were rinsed to remove non adherent cells and subsequently infected with 5 × 10^5^*A. fumigatus* conidia. The wells were then centrifuged at 125 × *g* for 10 min at 37°C to synchronize conidial exposure to the cells. At time points 0, ½, 1, 2, 4 and 8 h, the cell medium was removed and the infected cells were fixated in 4% formaldehyde containing 1% CaCl2 (w/v) in 0.121 M Na-cacodylate adjusted to pH 7 for 24 h. Then the cells were washed in Na-cacodylate buffer and preserved in the Na-cacodylate buffer. The samples were then divided into smaller portions (1 mm^2^) with a razor blade, dehydrated in graded ethanol solutions, and embedded in LX112 resin (Ladd Research, Burlington, Vermont, USA). Semithin sections (2 μm) were stained with toluidine blue to select the most appropriate regions for ultrathin sectioning. Ultrathin sections of 90 nm were cut with a diamond knife on a Reichert Jung Ultracut microtome (Jung, Vienna, Austria), mounted on formvar-coated copper grids, and stained with uranyl acetate and lead citrate. The samples were examined under a Jeol EXII transmission electron microscope (JEOL Ltd, Zaventem, Belgium) at 80 kV.

### Cytotoxicity assay

To determine the cytotoxic effect of *A. fumigatus* on avian respiratory macrophages, pigeon respiratory macrophages were seeded in 24-well plates. From each of 5 pigeons, one well with pigeon respiratory macrophages was exposed to 1 mL of 10^5^ conidia/mL and one well was left untreated. The plates were then centrifuged at 125 × *g* for 10 min. After 6 h of incubation, the wells were rinsed and exposed to ice cold ethidium monoazide (EMA) solution (50 μg/mL) for 20 min in the dark. The wells were then exposed to an incandescent lamp (45W) for 10 min. Next, the cells were rinsed and fixed with 4% (v/v) para-formaldehyde for 10 min at room temperature. After washing, the cells were stained with Hoechst (10 μg/mL) for 15 min at room temperature. Per well, 100 macrophages were analyzed for viability using fluorescence microscopy.

### Time-lapse video microscopy of the interaction of pigeon respiratory macrophages with *A. fumigatus* conidia

To verify intracellular germination and cellular lysis, pigeon respiratory macrophages (10^5^/mL) were seeded into a chamber slide system in sterile RPMI 1640 with glutamine medium supplemented with 10% foetal bovine serum and 1% penicillin/streptomycin and incubated overnight. After 2 h, the cells were rinsed three times with HBSS at 37°C. Cells were inoculated with 1 mL of 10^5^ conidia/mL in RPMI 1640 (+ 10% foetal bovine serum and 1% L-glutamine) and the plates were centrifuged at 125 × *g* for 10 min. The cells were allowed to ingest the conidia for 1 h. Medium containing non adherent, non phagocytosed conidia was removed, and wells were rinsed three times using HBSS with Ca^2+^-Mg^2+^. To discriminate between ingested and adherent conidia, the macrophages were further incubated with Calcofluor white M2R with a final concentration of 25 μM. After 30 min, the cells were washed and resuspended in RPMI 1640 (+ 10% foetal bovine serum and 1% L-glutamine). The chamber slide dishes were placed on an Olympus IX81 fluorescence microscope (Olympus, Zoeterwoude, The Netherlands) connected to a Cell*M imaging system (Olympus), and equipped with a Hamamatsu B/W Orca camera (Hamamatsu Photonics, Louvain-La-Neuve, Belgium), a Märzhaüser automated microscope table (Märzhaüzer Wetzlar Gmbh, Wetzlar, Germany), and a CellCubator climate chamber (Olympus). Bright-field images of 12 cells were collected every 15 min for 15 h.

### Statistical analysis

The differences in percentage of apoptotic and necrotic pigeon respiratory macrophages between controls and infected wells were statistically analyzed using a Two-sample T test.

## Results

The respiratory system of healthy pigeons showed notable fluctuations in the amount of free avian respiratory macrophages recovered after flushing. The yield of pigeon respiratory macrophages ranged between 3.2 × 10^4^ to 2.0 × 10^7^ with an average of 4.0 × 10^6^ +/- 7.4 × 10^6^ respiratory macrophages per pigeon.

The pigeon respiratory macrophages exhibited a moderate phagocytosis and limited killing capacity in the first hours post incubation. The fluorescence assay showed that of the conidia associated with the macrophages, on average, 25% +/- 1 were phagocytosed after 1 h in pigeon respiratory macrophages and the conidiacidal assay showed that on average, 16% +/- 6 of the macrophage-associated conidia were killed after 4 h.

**Figure 1:**
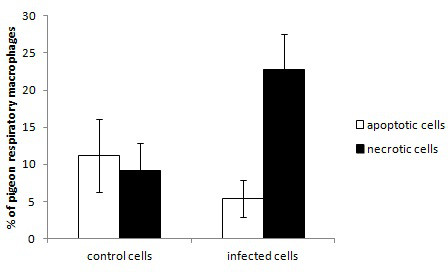
**Average percentage +/- standard deviation of apoptotic and necrotic pigeon respiratory macrophages after 6 h of incubation with or without*A. fumigatus*conidia.** Cell death was assessed using EMA and Hoechst staining and fluorescence microscopic evaluation of 100 macrophages. The experiment was done 5 times independently.

Pigeon respiratory macrophages exposed to *A. fumigatus* conidia exhibited significantly more necrotic (positive on EMA staining) (*P* < 0.01) and significantly less apoptotic (fragmented nucleus visualized using Hoechst staining) cells (*P* < 0.05) than control cells. In infected wells, on average 22.8% +/- 4.7 of the cells were necrotic and 5.4% +/- 2.5 were apoptotic whereas 9.2% +/- 3.7 were necrotic and 11.2% +/- 4.9 were apoptotic in the non-infected wells (Figure 1).

**Figure 2:**
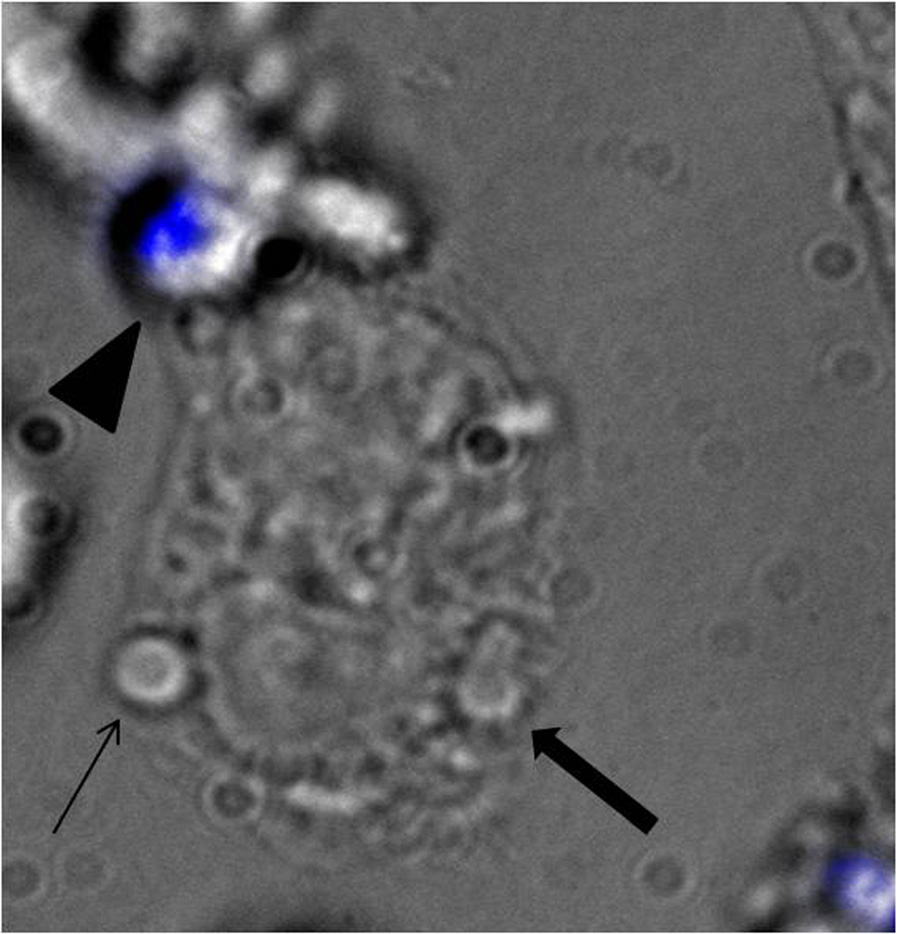
**A pigeon respiratory macrophage with a germinating conidia (thick arrow) and a non-germinating conidia (thin arrow).** An overlay is shown of the light microscopic image and the blue fluorescence of non-phagocytised conidia (filled arrowhead) (magnification 600×).

**Figure 3:**
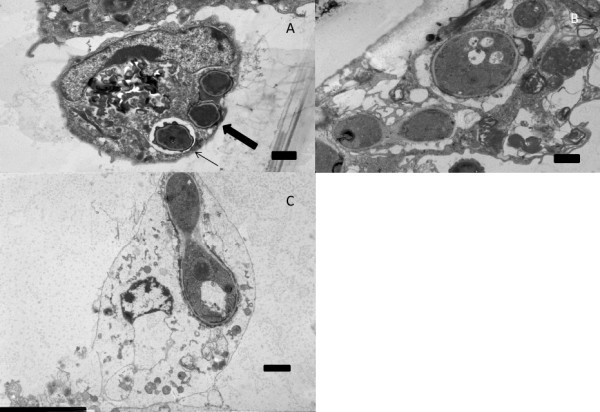
**Intracellular germination of conidia inside pigeon respiratory macrophages as revealed by transmission electron microscopy (A to C).** Panel A shows internalised conidia inside a vacuole (thin arrow) or inside the cytoplasm (thick arrow) of the macrophage after 1 h. Panels B and C show intracellularly germinating conidia inside a macrophage in an advanced state of degeneration **(B)** and in oncosis **(C)** after 8 h. Scalebars in all photos are 1 μm.

Intracellular germination in the cytoplasm of pigeon respiratory macrophages was observed in a small portion of these cells. Six hours after ingestion, the fluorescence assay demonstrated that intracellular germination of conidia occurred in 2% +/- 1 of the pigeon respiratory macrophages (Figure 2). TEM was performed to observe the intracellular trafficking of the conidia in macrophages and to verify that conidia can germinate within avian respiratory macrophages as observed in the fluorescence assay. After 1 h of contact between the conidia and the macrophages, some of the conidia were present in phagosomes in the cytoplasm of the macrophages (Figure 3A) while some of the conidia appeared to lay free in the cytoplasm of the cells (Figure 3A). Fusion of the phagosomes, containing conidia, with lysosomes was not observed. Autophagolysosomes and secondary lysosomes were present in multiple macrophages at different time points. Germtube-formation in the cytoplasm of the avian respiratory macrophages was observed 8 h after the initial contact (Figure 3B+C). These macrophages were in an advanced stage of degeneration (Figure 3B), leading to oncosis (Figure 3C). During time lapse video imaging, intracellular germination was observed and lysis of the cells was frequently observed after ingestion of multiple conidia (data not shown).

## Discussion

This is the first study that reports the phagocytic and killing capacity of avian respiratory macrophages against *A. fumigatus* conidia. The pigeon respiratory macrophages demonstrated a limited capacity of killing conidia in the first 5 hours post infection, compared to mammals [6,8-10]. However, germination was inhibited in more than 95% of the cell-associated *A. fumigatus* conidia, rendering the avian macrophage a highly efficient first line defense against *A. fumigatus*. Inhibition of germination was also observed in alveolar macrophages derived from mice, rabbits and humans [6]. Even though only a limited number of free respiratory macrophages is present in the avian respiratory tract, a rapid influx of activated avian respiratory phagocytes can provide an adequate protection against *A. fumigatus* infections in birds [22].

A small proportion of internalised conidia was still capable of germinating inside the avian macrophages. Intracellular germination was associated with degeneration, oncosis and necrosis of the macrophages. The damage to the macrophages may be elicited by gliotoxin. Gliotoxin is a toxin produced by *A. fumigatus* and is associated with cytotoxicity and cell death in murine macrophages [23]. Taking the limited number of resident respiratory macrophages into account, high environmental exposure to *A. fumigatus* conidia may overwhelm this defense mechanism, which perhaps may lead to colonization of the respiratory tract through intracellular germination of a limited number of conidia that caused macrophage cell death [16,22,24].

In conclusion, this study demonstrates that the majority of *A. fumigatus* conidia was either killed or their germination inhibited by avian respiratory macrophages. However large amounts of *A. fumigatus* conidia in the respiratory tract can result in intracellular germination and lysis of the phagocytic cells, which may contribute to colonization of the respiratory tract.

## Competing interests

The authors declare that they have no competing interests.

## Authors’ contributions

LVW, AM and FP participated in the design of the study. LVW, KDH, HF and SJL performed the experiments and LVW, RD, KDH, AM and FP analysed the data. LVW, AM, FP, RD and FH wrote the manuscript. All authors read and approved the final manuscript.
